# Functional *Ser205Leu* polymorphism of the nerve growth factor receptor (*NGFR*) gene is associated with vagal autonomic dysregulation in humans

**DOI:** 10.1038/srep13136

**Published:** 2015-08-17

**Authors:** Chuan-Chia Chang, Wen-Hui Fang, Hsin-An Chang, San-Yuan Huang

**Affiliations:** 1 Department of Psychiatry, Tri-Service General Hospital, National Defense Medical Center, Taipei, Taiwan; 2 Graduate Institute of Medical Sciences, National Defense Medical Center, Taipei, Taiwan; 3 Department of Family and Community Medicine, Tri-Service General Hospital, National Defense Medical Center, Taipei, Taiwan

## Abstract

Evidence indicates that reduced cardiac vagal (parasympathetic) tone, a robust cardiovascular risk factor, is a trait vulnerability marker of major depressive disorder (MDD). The *Ser205/Ser205* genotype of the functional polymorphism (*Ser205Leu*) of the nerve growth factor receptor (*NGFR*), also called p75 neurotrophin receptor (*p75*^*NTR*^), gene is reported to increase the risk of MDD. Here, we hypothesized that the *NGFR Ser205Leu* polymorphism may have an effect on vagal control. A sample of 810 healthy, drug-free, unrelated Han Chinese (413 males, 397 females; mean age 35.17 ± 8.53 years) was included in the *NGFR* genotyping. Short-term heart rate variability (HRV) was used to assess vagus-mediated autonomic function. Potential HRV covariates, such as mood/anxiety status and serum metabolic parameters, were assessed. Homozygotes of the *Ser205* allele had significantly lower high frequency power and root mean square of successive heartbeat interval differences, both HRV indices of vagal modulation, compared to *Leu205* allele carriers. Even after adjusting for relevant confounders, these associations remained significant. Further stratification by sex revealed that the associations were observed only in males. Our results implicate that decreased parasympathetic activity is associated with the *NGFR Ser205/Ser205* genotype in a gender-specific manner, suggesting a potential role of *NGFR* polymorphism in modulating cardiac autonomic function.

Heart rate variability (HRV), a non-invasive electrocardiographic (ECG) index enabling quantitative assessment of autonomic nervous system (ANS) activity^1^, has been shown to play a critical role in the development of mood disorders. In a recent meta-analysis of major depressive disorder (MDD) and HRV, Kemp *et al.*^2^ reveal that unmedicated depression without cardiovascular disease (CVD) is associated with decreased vagal (parasympathetic) tone, as indexed by reduced HRV. Furthermore, research has shown that despite resolution of depressive symptoms, the low HRV in patients with MDD is not resolved, indicating that reduced vagal tone is a trait vulnerability marker of MDD^3^.

Epidemiologic studies have demonstrated that MDD is highly related to CVD, both of which are leading burdens of disease worldwide. While MDD increases the risk of CVD by approximately 2.7-fold^4^, up to 50% of CVD patients suffer from depression^5^. Moreover, patients with MDD have a three- to four-fold increased risk of cardiac mortality, irrespective of cardiac disease history^6^. Since reduced HRV is a known predictor of cardiovascular morbidity and mortality^7^,^8^, vagal dysregulation has been further posited as the link of MDD to adverse cardiovascular events^9^,^10^.

The nerve growth factor receptor (NGFR), also known as p75 neurotrophin receptor (p75^NTR^), which binds to all the members of the neurotrophin family, has an essential role in eliciting neuronal apoptotic signaling^11^ and in suppressing synaptic plasticity^12^. NGFR has been involved in modulating the synaptic transmission of acetylcholine^13^,^14^, a key neurotransmitter of the parasympathetic nervous system. Furthermore, patients with MDD and those who committed suicide exhibit increased levels of NGFR mRNA/proteins^15^,^16^. An altered presence of NGFR has also been implicated in CVD and related disorders^17^.

A missense single nucleotide polymorphism (727*C > T*; NCBI dbSNP ID: *rs2072446*) in the *NGFR* gene causes an allele change at position 739 in the mRNA (NCBI accession number: NM_002507.3) and subsequently, a serine to leucine substitution at the amino acid position 205 (*Ser205Leu*) (NCBI accession number: NP_002498.1), thereby altering NGFR function. Homozygotes of the *Ser205* allele exhibit higher plasma NGFR protein levels^18^. A recent meta-analysis of 1060 patients and 1688 controls demonstrates that the *Ser205*/*Ser205* genotype of *NGFR* confers susceptibility to MDD^19^. However, no study has investigated the correlation between the functional *Ser205Leu* polymorphism and cardiac vagal control. Thus, investigating the influence of *NGFR Ser205Leu* polymorphism on HRV may be crucial for understanding the mechanism of vagal modulation and providing possible insights into the complex pathophysiology of MDD and cardiovascular co-morbidities.

Many non-genetic factors like age, sex, cigarette smoking, physical activity, position, hour of the day, medications, and medical and psychiatric morbidities influence HRV^20^. Studying drug-free healthy subjects in a well-controlled manner can therefore minimize these confounding factors to reveal more precisely the *NGFR* gene effect on vagal control. Using HRV measures in a large sample of healthy medication-free Han Chinese subjects, the present study tested the hypothesis that the *Ser205Leu* polymorphism of *NGFR* may have a modulatory effect on vagal function and whether this association remained significant after adjusting for relevant confounders.

## Results

### Demographics and clinical characteristics of the *NGFR* genotype groups

A total of 810 healthy subjects (413 males, 397 females; mean age: 35.17 ± 8.23 years) were selected for the study. The demographic and clinical data for all the subjects with the *NGFR* (*Ser205Leu*) genotype are listed in Table 1. The *NGFR* genotype distribution in the present sample was in Hardy-Weinberg equilibrium. The *NGFR* genotype groups did not differ in demographic data and clinical characteristics, e.g., age, Beck Anxiety Inventory (BAI) scores, and systolic and diastolic blood pressure, except in serum triglyceride level, which had a trend of significance (*p *= 0.07) (Table 1).

### Non-genetic factors associated with vagal indices of HRV

The associations between vagal indices of HRV and confounding variables are summarized in Table 2. Women had significantly reduced high frequency power (HF) and the root mean square of successive R-R interval differences (RMSSD) than men. Habitual physical activity positively correlated with both the vagus-mediated HRV indices. In contrast, age and serum total cholesterol, triglyceride, and fasting glucose were negatively related to at least one vagal index of HRV. Furthermore, scores for BAI and Beck Depression Inventory-II (BDI), smoking status, and body mass index (BMI: kg/m^2^) had no correlation with any HRV variable (Supplementary Table 1).

### Association of *NGFR* genotypes with vagus-mediated HRV indices

**Figure 1:**
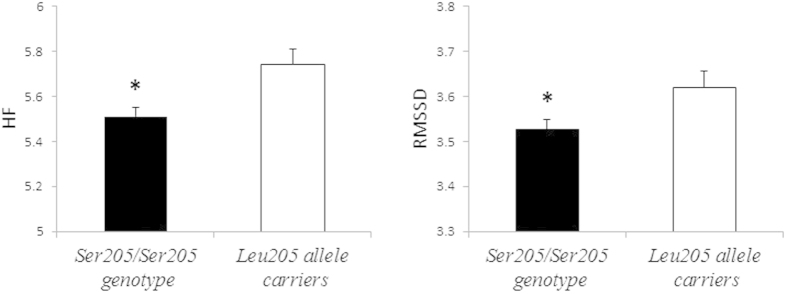
Unadjusted effects of the *NGFR* genotype on vagal indices of heart rate variability. HF, high frequency power (ln[ms^2^]); RMSSD, the root mean square of successive heartbeat interval differences (ln[ms]); *p < 0.05.

The relationship among the vagus-mediated HRV indices and the *NGFR Ser205Leu* genotypes are presented in Fig. 1. Participants with the *NGFR Ser205/Ser205* genotype showed significantly reduced HF (t = 2.56, *p *= 0.011) and RMSSD (t = 2.13, *p *= 0.034) compared to *Leu205* carriers.

All of the HRV-associated factors were then entered as covariates into the ANCOVA, with HRV indices as dependent variables. After adjusting for relevant covariates, including age, sex, physical activity, and serum metabolic measures, the aforementioned associations with HF and RMSSD persisted and became more robustly significant (F = 10.79, *p *= 0.001 and F = 7.28, *p *= 0.007, respectively) (Table 3). Further analysis with stratification by sex, there was a significant *NGFR* genotype effect on HF (F = 13.29, *p *< 0.001) and RMSSD (F = 7.69, *p *= 0.006) in men, but not in women (HF: F = 0.48, *p *= 0.49; RMSSD: F = 0.65, *p *= 0.42) (Table 4).

## Discussion

The key finding of the present study is that *NGFR Ser205Leu* polymorphism exerts a robust effect on vagal autonomic function. Even after adjusting for known covariates, subjects with the *Ser205*/*Ser205* genotype (high-expression allele homozygotes) still have significantly lower HF and RMSSD, indicating reduced vagal activity, compared to subjects bearing at least one *Leu205* allele. Post-hoc analysis stratified by sex reveals that these associations are observed only in men. To date, this is the first study to investigate the role of the functional *NGFR* genetic variant in human vagal modulation.

The findings have several implications. First, heritability studies already demonstrate that genetic determinants contribute substantially to variance in vagus-mediated HRV^21^. A growing body of evidence suggests that NGFR may have modulating effects on vagal control. Anatomic researches reveal that the NGFR is present in neurons and satellite cells of parasympathetic ganglia^22^,^23^. Studies have also shown that neurotrophins like brain-derived neurotrophic factor (BDNF) and nerve growth factor, acting through NGFR, can lead to altered neuronal cholinergic phenotypes^13^,^14^. Furthermore, choline acetyltransferase, the enzyme synthesizing acetylcholine, is known to be activated by BDNF through a NGFR dependent pathway^24^. The findings here, focusing on a study of humans, complement previous studies and demonstrate that the functional *NGFR* genetic variant plays an important role in vagal regulation.

Second, ANS is highly adaptable and allows the organism to maintain its balance when experiencing strain or stress. In contrast, a rigid and inflexible system can lead to somatic and psychological pathologies^10^,^25^. Decreases in HRV characterized as reduced vagal activity is associated with the risk of developing future CVD like angina pectoris, myocardial infarction, coronary heart disease, or congestive heart failure, and may increase cardiac mortality in patients with existing heart disease^7^,^26^. Furthermore, findings from several studies indicate that NGFR is involved in the development of CVD, such as coronary atherosclerosis, myocardial infarction, congestive heart failure, and sudden cardiac death^27^,^28^,^29^. Taken together, the reduced cardiac vagal modulation observed in the healthy individuals with the “high-expressing” *Ser205*/*Ser205* genotype of *NGFR* may show an underlying pathway explaining why NGFR increases susceptibility to the onset of CVD.

Third, the *Ser205/Ser205* genotype of the *NGFR* has been associated with the prevalence of MDD^19^,^30^. However, such an association has not been demonstrated in a study with a relatively small sample size^31^. Considering the inconsistency of these results, an analysis of HRV providing quantitative markers of ANS function^32^ to understand the physiologic role of the studied *NGFR* variant may complement conventional case-control studies. Since low vagal tone is associated with MDD^2^,^3^ and NGFR has been shown to affect depression^15^,^33^, the present results raise the possibility that subjects with the *NGFR Ser205/Ser205* genotype have increased risk of developing MDD in combination with vagal withdrawal. Given that the results of this study is from a non-clinical cohort, reduced vagal modulation may be an endophenotype of individuals with a genetic susceptibility (i.e., the *Ser205/Ser205* genotype of *rs2072446*) for MDD rather than a consequence of the development of the illness.

Fourth, evidence suggests that NGFR induced apoptotic signaling plays an important role in the pathophysiology of Alzheimer’s disease (AD)^34^, which is characterized by basal forebrain cholinergic neuronal dysfunction resulting in profound memory disturbances and irreversible impairment of cognitive function. Recently, the functional *Ser205Leu* variant of *NGFR* has been reported as a risk factor for familial AD^35^. Moreover, a study has also shown that among apolipoprotein E (*ApoE*) *ε4* allele non-carriers, the *NGFR* polymorphism increases the risk of AD, which is affected by the presence of type 2 diabetes mellitus^36^. Consistent with these reports, our finding that the *NGFR Ser205Leu* variant is involved in vagal (cholinergic) neuronal activity of HRV suggests that the *NGFR* polymorphism may play an important role in modulating cholinergic neuronal phenotypes.

Fifth, in the present study, women exhibited significantly lower HF and RMSSD than men. This may be because the women were older than the men (mean age: 36.81 ± 8.79 [range: 20–60] *vs.* 33.60 ± 7.98 [range: 20–61] years; *p *< 0.001), and age is strongly negatively related to vagus-mediated HRV. Indeed, there was no significant gender difference in vagal indices of HRV when adjusted for age (data not shown, *p *> 0.05).

Interestingly, when stratified by sex, we observed that the *NGFR* gene effects on the vagal indices of HRV were significant in men, but not in women. Experimental studies have shown that exogenous estrogen administration attenuates the expression of *NGFR* in cholinergic neurons of mouse^37^,^38^. Most of the females enrolled in this study were of reproductive age, and therefore it is possible that estrogen may have reduced the expression efficiency for women carrying the “high-expressing” *NGFR Ser205/Ser205* genotype. This may explain the sexually dimorphic effects of the *NGFR* gene on vagal autonomic control.

From a previous study, the parasympathetic activity of post-menopausal women is reportedly lower than that of pre-menopausal women^39^. Vagal activity is also reduced in the luteal phase than in the follicular phase of the female menstrual cycle^40^. Thus, the finding that parasympathetic modulation in females does not differ significantly among the studied *NGFR* genotypes should be interpreted cautiously, because this study did not check the menstrual status/estrogen concentration of the female participants. Recently, Fuji *et al.*^19^ have reported that *NGFR Ser205* homozygotes are associated with increased risk of MDD in a relatively older female subgroup (median age, 54 years). Future studies testing whether changes in estrogen level (e.g., post-menopausal) have an impact on the “high-expressing” *NGFR Ser205/Ser205* genotype contributing to a decreased vagal modulation compared to *Leu205* allele carriers in women are warranted.

Lastly, many non-genetic confounders like age, sex, physical activity, and serum metabolic profiles, have effects on the vagal indices of HRV (Table 2). Different methods of data processing by various authors often elicit conflicting HRV results^20^. Nonetheless, the present study has carefully controlled for confounding factors. All of the participants are also medication-free and have undergone structured psychiatric evaluation and medical health check-up to exclude illnesses. Moreover, since ethnic stratification among study samples may lead to resetting population HRV patterns^41^, all of the subjects in the present study are unrelated Han Chinese subjects drawn from a genetically homogeneous population pool in Taiwan^42^. Thus, this study may precisely reveal the *NGFR* gene effect, without ethnic stratification bias, on autonomic vagal modulation. Taken together, there will be less likelihood of producing a false-positive result.

This study has several limitations. The study utilized cross-sectional data only. As such, the long-term impact of the *NGFR* polymorphism on ANS modulation cannot be directly inferred. Furthermore, the reliability of using short-term HRV recoding may be a concern. Nonetheless, it has been shown to be reliable especially when short-term HRV is measured in healthy subjects at rest^43^. Another limitation is that the respiratory rate has not been controlled even as this has been shown to influence the vagal indices of HRV in the clinical settings^20^. Fortunately, this may not affect the findings because differences in HF between spontaneous and metronome-guided breathing are extremely small in healthy subjects^44^. Moreover, only a single *NGFR* polymorphism was examined. Future work should consider examining other genetic variants and other promising candidate genes, such as choline transporter^45^ so that additive and interactive effects can be explored. Lastly, since this is the first study on this matter, replication conducted in an independent sample is needed to corroborate the present results.

In conclusion, this study provides initial evidence that *NGFR Ser205Leu* polymorphism modulates the autonomic vagal outflow to the heart, particularly in men. A longitudinal follow-up study investigating the impact of the *NGFR* variant-associated vagal withdrawal on the incidence of MDD and CVD should be conducted in the future.

## Methods

### Participants

The study cohort was composed of volunteers who underwent annual health examinations at the Tri-Service General Hospital, a medical teaching hospital of the National Defense Medical Center in Taipei, Taiwan. All were biologically unrelated Han Chinese subjects. After detailed questionnaire screening, those taking any medication for at least one month prior to the start of the study or those with a personal history of medical diseases (e.g., syncope or orthostatic hypotension), psychiatric illnesses, substance dependence, or pregnancy were excluded. After initial screening, 985 adult Han Chinese were recruited. The demographic data collected included age, sex, BMI, smoking status (yes/no), and weekly exercise level (none/1–2 times per week/≥3 times per week).

The institutional review board of the Tri-Service General Hospital approved the study protocol (TSGHIRB: 1-101-05-089), which adhered to the guidelines of the Declaration of Helsinki. All of the participants provided written informed consent.

### Assessment of psychiatric morbidity and mood and anxiety levels

Each enrolled subject was further evaluated by a trained research assistant using the Chinese version of the Mini-International Neuropsychiatric Interview (MINI), a structured diagnostic instrument based on the Diagnostic and Statistical Manual of Mental Disorders, Fourth Edition (DSM-IV) criteria^46^. Participants with mental illness were excluded while the rest were further evaluated for their anxiety/mood status.

The Chinese version of the BAI, a self-rated 21-item scale, was used to measure the intensity of anxiety in the past week^47^. Each item was rated on a four-point scale, ranging from ‘not at all’ (0) to ‘severely’ (3). The total scores ranged from 0–63, with higher scores indicating higher anxiety. The mood status was assessed with the Chinese version of the BDI, a 21-item questionnaire assessing self-reported levels of depression over the preceding two weeks^48^. Each question was assigned a score of 0–3, with 3 indicating the most severe depressive features (total score range, 0–63). Higher total scores correlated with more severe depression. Both the Chinese BAI and BDI were highly reliable and valid^47^,^48^. Subjects with higher levels than a mild degree of anxiety (BAI > 15) or depression (BDI > 19) were also excluded.

### Assessment of medical conditions

All of the study participants received health check-ups, including physical examination, biochemical (blood, urine, and stool specimens) analysis, and chest X-ray and electrocardiogram examinations. Systolic and diastolic blood pressures were measured. Fasting plasma glucose was determined by the glucose oxidase method, while triglyceride and total cholesterol levels were measured using the dry, multi-layer analytical slide method^49^.

Participants with organic diseases like cardiovascular diseases (e.g., hypertension, arrhythmia), metabolic disorders (e.g., hypercholesterolemia, hypertriglyceridemia, and diabetes mellitus), liver or kidney diseases, malignancy, neuropathy, or obesity (BMI ≥ 30 kg/m^2^) were excluded.

### Measurements of heart rate variability

Heart rate variability was measured using an electrocardiogram analyzer (SA-3000P; Medicore Co., Ltd., Korea) to acquire, store, and process ECG signals^50^. All of the subjects were examined in a quiet room with standard temperature (22–24 °C). To accommodate diurnal fluctuation, ECG monitoring was done in the morning (08:00 AM–12:00 noon). After sitting at rest for 15 min, each participant underwent ECG recording with normal breathing in a sitting position for 5 min. The average HR (beats/min) was derived from the R waves of the ECG.

The system automatically analyzed the changes in heart rate using time domain and frequency domain analyses^1^. The RMSSD was used to compare the time domain index (set as milliseconds) and reflected cardiac parasympathetic activity. Power spectral analysis was performed using fast-Fourier transformation and the power spectrum was quantified into standard frequency domain measurements. Vagal control of the spectral HRV was represented by HF (0.15–0.4 Hz). All HRV measurements were logarithmically transformed to correct the skewed distribution.

### Laboratory genotyping

From blood samples, DNA was isolated for genotyping using a QIAamp^®^ DNA blood kit according to the manufacturer’s instructions (Qiagen^®^, Valencia, CA). The quality of isolated genomic DNA was assessed for each sample by agarose gel electrophoresis, while the quantity of DNA was determined by spectro-photometry (NanoDrop^®^, Wilmington, USA). The studied *NGFR Ser205Leu* polymorphism (*rs2072446*) was genotyped by the TaqMan^®^ 5′-exonuclease assay using an Applied Biosystems^®^ (ABI^®^, CA, USA) Prism 7900 instrument, as described previously^19^.

### Statistical analysis

The allele and genotype frequencies of the *NGFR* gene were calculated and genotypic distribution was compared to the predicted values from the Hardy-Weinberg equilibrium. Because the number of homozygotes for the *Leu205* allele was small (*n *= 7), *Leu205* allele carriers were compared to the *Ser205* allele homozygotes. The χ^2^ statistics were used to compare categorical variables while the Student *t* test was used for continuous variables among the *NGFR* genotype groups.

To control for non-genetic confounders, the association between vagus-mediated HRV indices and demographic/clinical variables (i.e., age, sex, BAI, BDI, BMI, smoking status, physical exercise levels, and serum metabolic parameters) were tested. Pearson’s correlation was used to evaluate the relationship among variables with normal distribution, while Spearman’s correlation was used for non-normal distribution. Variables associated with HRV were then used as covariates in separate ANCOVA models testing the effects of genotype on each of the HRV indices. Post-hoc analysis stratified for sex was further performed. Statistical significance was set at *p *< 0.05 (two-tailed). All statistical analyses were conducted using the statistical package SPSS (Version 15.0; SPSS, Taipei, Taiwan).

Power calculations were conducted using the Quanto software version 1.2.4^51^. The total sample (*n *= 810) had a power of 99.9% for detecting a proportion of variance, *R*^2^ = 0.05, in HRV indices explained by gene effects. Furthermore, the powers of the HRV indices explained by genetic effects in males (*n *= 413) and females (*n *= 397) were 99.6% and 99.5%, respectively.

## Additional Information

**How to cite this article**: Chang, C.-C. *et al.* Functional *Ser205Leu* polymorphism of the nerve growth factor receptor (*NGFR*) gene is associated with vagal autonomic dysregulation in humans. *Sci. Rep.* **5**, 13136; doi: 10.1038/srep13136 (2015).

## Supplementary Material

Supplementary Table 1

## Figures and Tables

**Table 1:** Demographic data and clinical characteristics of the study participants stratified by *NGFR* genotype.

Characteristics	*NGFR* genotypes			
	*Ser205/Ser205* genotype (*n* = 636)	*Leu205* allele carriers (*n* = 174)	t or χ^2^	*p*
Age (year)	35.05 ± 8.48	35.63 ± 8.75	0.80	0.43
Female/Male, *n* (%)	315/321 (49.5/50.5)	82/92 (47.1/52.9)	0.32	0.61
BMI (kg/m^2^)	22.55 ± 3.21	22.78 ± 3.07	0.86	0.39
Current smoker, *n* (%)	97 (15.3)	29 (16.7)	0.21	0.64
Weekly regular exercise			0.14	0.93
Nil, *n* (%)	360 (56.6)	98 (56.3)		
1–2 times/week, *n* (%)	179 (28.1)	51 (29.3)		
≥3 times/week, *n* (%)	97 (15.3)	25 (14.4)		
Heart rate (beats/min)	72.26 ± 12.19	70.66 ± 9.91	1.60	0.11
SBP (mm Hg)	112.44 ± 12.83	111.07 ± 12.74	1.24	0.22
DBP (mm Hg)	72.81 ± 9.14	71.48 ± 9.71	1.67	0.10
Total cholesterol (mg/dl)	174.72 ± 28.45	175.03 ± 27.29	0.13	0.90
Triglyceride (mg/dl)	87.99 ± 38.74	94.07 ± 39.55	1.82	0.07
Fasting glucose (mg/dl)	85.94 ± 7.21	86.92 ± 7.99	1.54	0.12
BAI (score)	4.06 ± 4.39	4.49 ± 4.60	1.22	0.22
BDI (score)	5.42 ± 4.81	5.68 ± 5.08	0.63	0.53

Continuous variables are reported as mean ± standard deviation; categorical variables as column sum (percentage).

Abbreviations: BAI, Beck Anxiety Inventory; BDI, Beck Depression Inventory-II; BMI, body mass index; DBP, diastolic blood pressure, SBP, systolic blood pressure.

**Table 2:** Confounding variables associated with vagus-mediated HRV indices among the study subjects.

HRV indices	Age	Sex (female/male)	Physical activity (none/low/high)	Total cholesterol	Triglyceride	Fasting glucose
HF	−0.38^***^	0.13^***^	0.08^***^	−0.06^**^	−0.14^***^	−0.15^***^
RMSSD	−0.35^***^	0.12^***^	0.14^***^	−0.10^**^	−0.15^***^	−0.17^***^

First category in parenthesis is the reference group.

Physical activity levels were classified as none, 1–2 times per week (low), or ≥3 times per week (high).

Abbreviations: HF, high frequency power (ln[ms^2^]); HRV, heart rate variability; RMSSD, the root mean square of successive heartbeat interval differences (ln[ms]). ^*^p < 0.05. ^**^p < 0.01. ^***^p < 0.001.

**Table 3:** Adjusted means of vagus-mediated HRV indices presented by *NGFR* genotypes.

HRV indices	*NGFR* genotypes		F	*p*
	*Ser205*/*Ser205* genotype (*n* = 636)	*Leu205* allele carriers (*n* = 174)		
HF†	5.50 (0.04)	5.77 (0.07)	10.79	**0.001**
RMSSD†	3.52 (0.02)	3.63 (0.04)	7.28	0.007

Data are shown as mean (standard error).

Abbreviations: HF, high frequency power (ln[ms^2^]); HRV, heart rate variability; RMSSD, the root mean square of successive heartbeat interval differences (ln[ms]).

^†^Adjusted for covariates: age, sex, physical activity, and serum metabolic measures.

**Table 4:** Adjusted means of heart rate variability (HRV) indices presented by *NGFR* genotypes and sex†.

HRV indices	Female sub-group		F	*p*	Male sub-group		F	*p*
	*Ser205*/*Ser205* genotype (*n* = 315)	*Leu205* allele carriers (*n* = 82)			*Ser205*/*Ser205* genotype (*n* = 321)	*Leu205* allele carriers (*n* = 92)		
HF†	5.40 ± 0.05	5.48 ± 0.10	0.48	0.49	5.60 ± 0.05	6.00 ± 0.10	13.29	**.0.001**
RMSSD†	3.47 ± 0.03	3.52 ± 0.05	0.65	0.42	3.58 ± 0.03	3.73 ± 0.05	7.69	**0.006**

Data are presented as mean ± standard error.

Abbreviations: HF, high frequency power (ln[ms^2^]); RMSSD, the root mean square of successive heartbeat interval differences (ln[ms]).

^†^Adjusted for the covariates: age, physical activity, and serum metabolic measures.
