# Supplementary material for: Functional Ser205Leu polymorphism of the nerve growth factor receptor (NGFR) gene is associated with vagal autonomic dysregulation in humans
Source: Sci Rep. 2015 Aug 17;5:13136. doi: 10.1038/srep13136 (PMC4538378; doi:10.1038/srep13136)
Supplement: Supplementary Table 1 [file srep13136-s1.doc]

**Supplementary information**

**Manuscript number: SREP-15-05316A**

**Title of the manuscript:**

Functional *Ser205Leu* polymorphism of the nerve growth factor receptor (*NGFR*) gene is associated with vagal autonomic dysregulation in humans

**Author list:**

Chuan-Chia Chang, Wen-Hui Fang, Hsin-An Chang, and San-Yuan Huang

**Supplementary Table 1. Non-genetic factors not associated with vagal indices of HRV among the study participants**

|  | BAI | BDI | Smoking status  (No/yes) | BMI |
| --- | --- | --- | --- | --- |
| HRV indices |  |  |  |  |
| HF | -0.07 | 0.00 | -0.02 | 0.00 |
| RMSSD | -0.07 | 0.00 | 0.02 | -0.02 |

First category in parenthesis is the reference group.

Abbreviations: BAI, Beck Anxiety Inventory; BDI, Beck Depression Inventory-II; BMI, body mass index (kg/m^2^); HRV, heart rate variability; HF, high frequency power (ln[ms^2^]); RMSSD, the root mean square of successive heartbeat interval differences (ln[ms]).

None of the correlation tests reached a significant level (all *p*>0.05).
